# Effects of sodium butyrate on the morphology, antioxidant capacity, mitochondrial integrity, and the transcriptome of the jejunum in early weaned lambs

**DOI:** 10.3389/fvets.2026.1768380

**Published:** 2026-06-23

**Authors:** Fangfang Zhao, Yingxi Tian, Wenhao He, Wanxin Tian, Ruonan Fan, Aizhong Zhang

**Affiliations:** College of Animal Science and Veterinary Medicine, Heilongjiang Bayi Agricultural University, Daqing, Heilongjiang, China

**Keywords:** antioxidant capacity, mitochondrion, sodium butyrate, transcriptome, weaned lamb

## Abstract

The aim of this study was to investigate the effects of sodium butyrate on the morphology, antioxidant capacity, mitochondrial integrity, and transcriptome in the jejunum of early weaned lambs. Ten 21-day-old healthy weaned lambs with an average weight of (6.53 ± 0.17) kg were selected for the experiment and randomly divided into a control group (CON) and a sodium butyrate group (NaB). The experimental lambs were fed with basic diet (CON) and experimental diet (NaB, supplement with 3 g/kg of coated sodium butyrate in the basic feed) for 21 days. The results showed that the addition of sodium butyrate significantly increased villus height (*p* < 0.05) and total antioxidant capacity (*p* < 0.05) in the jejunum of lambs, significantly reduced malondialdehyde content (*p* < 0.05) and the accumulation of reactive oxygen species (*p* < 0.05) in jejunum tissue, and maintained the integrity of mitochondrial structure. The transcriptome analysis of jejunal tissue showed that the addition of sodium butyrate into weaned lambs’ diets significantly altered the expression of genes and pathways related to jejunal metabolism (*p* < 0.05). In summary, the addition of sodium butyrate improved the intestinal health of early weaned lambs and maintained intestinal metabolic function. The research results provide a foundation for the application of sodium butyrate to improve intestinal health of lambs during early weaning phase.

## 1 Introduction

In modern sheep farming systems, artificial weaning interventions are commonly applied to suckling lambs to facilitate multiple births in ewes and promote rapid growth and development in lambs (1). This practice aims to shorten the lamb production cycle, enhance productivity, reduce feeding costs, and maximize economic benefits in sheep farming (2). During the artificial weaning process, lambs face the transition from breastfeeding to solid feed, which can be challenging. Their immature intestinal development and incomplete colonization of gut microbiota make them susceptible to intestinal epithelial damage due to the change in feed form (3). Additionally, lambs must adapt to rapid changes in their living environment and social interactions within the flock (4). These physiological and environmental changes triggered by weaning can lead to stress responses. Excessive oxidative stress resulting from these responses can further damage mitochondrial function within cells, affecting nutrient absorption and immune system health in lambs, ultimately compromising their production performance (5). Thus, it is essential to develop comprehensive strategies to mitigate the stress response associated with lamb weaning.

Sodium butyrate can serve as an energy source for intestinal epithelial cells and plays a critical role in maintaining gut health, regulating immune function, and preventing oxidative stress (6). Oxidative stress-induced intestinal damage typically manifests as compromised intestinal barrier function, increased intestinal cell apoptosis, and dysbiosis of gut microbiota (7). Furthermore, oxidative stress leads to mitochondrial damage by directly affecting mitochondrial DNA, proteins, and lipids (8). Recent research has shown that sodium butyrate improves gut health by modulating the proliferation and differentiation of intestinal epithelial cells (9). Additionally, sodium butyrate activates PGC1-alpha to enhance mitochondrial function (10). Moreover, sodium butyrate upregulates the expression and activity of antioxidant enzymes, increasing cellular antioxidant capacity and ultimately mitigating oxidative stress-induced damage to mitochondria and intestinal function (11). Consequently, based on the potential of sodium butyrate to enhance animal antioxidant capacity and improve gut function, this study aims to investigate whether the addition of sodium butyrate into early-weaned lamb diets can improve lamb intestinal morphology, antioxidant capacity, mitochondrial damage, and epithelial metabolism. These findings may provide valuable insights for the application of sodium butyrate in promoting lamb intestinal health during their crucial growth phase.

## 2 Materials and methods

### 2.1 Animals ethics statement

All animal experiments adhered to the animal experiment policy of the Animal Care Institution and Ethics Committee of Heilongjiang Bayi Agricultural University (Daqing, China; Grant No.: DWKJXY2022019).

### 2.2 Animals, experimental design and sample collection

Ten 21-day old healthy weaned lambs (South African Meat Merino × small-tailed Han sheep) with the similar weight (6.53 ± 0.17 kg) were selected for this trial. The weaned lambs were randomly divided into two groups with five replicates in each group. The whole trial lasted for 21 days. The experimental weaned lambs were fed with the basal diet (CON) and the basal diet with 3 g/kg coat sodium butyrate supplementation (NaB), respectively. Coated sodium butyrate was purchased from Dongying Herunde Biological Technology Co., Ltd. The product contained 30% sodium butyrate and was mainly fat-coated to achieve slow release of sodium butyrate. During the trial period, all weaned lambs were housed in slatted wood floor pens, individually. All pens equipped with a drinking bowl and feed chute. The diets were offered in equal amounts twice a day at 08:00 and 18:00, with ad libitum access to feed and fresh water for all lambs. The ingredient and nutrient composition of the basal diet was showed in Table 1.

**Table 1:** The ingredient and nutrient composition of the basal diet (DM basis) (%).

Items	Basal diet
Ingredients (%)	
Alfalfa	7.00
Oat Hay	5.00
Corn	45.00
Soybean meal	20.50
Wheat bran	8.00
Corn germ meal	10.00
Ground limestone	2.00
CaHPO_4_	0.50
Salt	0.50
NaHCO_3_	0.50
Premix^1^	1.00
Total	100.00
Nutrient levels^2^	
DM (%)	89.10
DE (MJ/kg)	13.87
CP (%)	21.52
EE (%)	2.58
NDF (%)	18.93
ADF (%)	8.21
Calcium (%)	0.86
Phosphorus (%)	0.59

^1^ The premix provided a per kg of diet as follows, Fe 25 mg, Cu 8 mg, Zn 40 mg, Mn 40 mg, I 0.3 mg, Se 0.2 mg, Co 0.1 mg, vitamin A 940 IU, vitamin E 20 IU. ^2^ DE was a calculated value, while others were measured values. DM, dry matter; DE, digestive energy; CP, crude protein; EE, ether extract; NDF, neuter detergent fibers; ADF, acidic detergent fibers.

At the end of the trial, lambs were stunned electrically at a voltage of 125 V for 10 s (electrodes applied on both sides of the head, behind the ears) and subsequently exsanguinated. Jejunal tissue samples were then collected, cleaned with sterile PBS to remove digesta. The middle segment of jejunal tissue was collected. A portion of the segment was stored at −80 °C for subsequent determination of antioxidant indices and transcriptome analysis. Another portion was fixed in 4% paraformaldehyde for observation of jejunal morphology and detection of ROS levels. The remaining tissues were preserved in 2.5% glutaraldehyde at 4 °C for transmission electron microscopy observation.

### 2.3 Jejunal antioxidant capacity measurments

The collected jejunal tissue was lysed and homogenized. Subsequently, the sample was centrifuged at 3000 rpm at 4 °C for 15 min, and the supernatant was harvested for analytical determination. The jejunal antioxidant indicators, including total antioxidant capacity (T-AOC), malondialdehyde (MDA) contents, superoxide dismutase (SOD) activity, and glutathione peroxidase (GSH-Px) activity, were measured using the commercial kits (Nanjing Jiancheng Bioengineering Research Institute, Nanjing, China) in accordance with the manufacturer’s protocols.

### 2.4 Jejunal histology

Paraformaldehyde-fixed jejunal samples were dehydrated and embedded in paraffin wax. Segments of cross-sections were cut at 5 μm thickness and stained with hematoxylin and eosin. The morphological structure of villus height and crypt depth was acquired by a microscope using an image processing and analysis system (Version 1, Leica Imaging Systems Ltd., Cambridge, United Kingdom). A total of 15 intact, well-oriented crypt-villus units were measured in jejunal tissue from each lamb. Villus height and crypt depth were blindly measured using an image processing and analyzing system (version 6.0, Image-Pro Plus).

### 2.5 Transmission electron microscopy of mitochondria

Approximately 1 mm^3^ jejunal tissues were collected from preserved samples and rinsed with 0.1 M PBS buffer (pH 7.4) three times for 15 min each time. Afterwards, the tissues were fixed with 1% osmic acid at room temperature. After gradient dehydration, the samples were subjected to sequential infiltration with mixed solutions of acetone and epoxy resin at volume ratios of 2:1, 1:1, and pure epoxy resin respectively, and incubated at 37 °C for 12 h. The permeated samples were put into the embedding plate, added with the embedding agent epoxy, and polymerized for 48 h in a 60 °C incubator. The specimen was sectioned using a LEICA UC7 ultratome (Leica UC7) and the sections were stained with 2% uranium acetate saturated aqueous solution and lead citrate for 15 min, respectively. The sections were dried at room temperature overnight. Transmission electron microscope (Tecnai G2 F20 S-TWIN, FEI Company, United States) was used for observation.

### 2.6 Transcriptomics profiling

Jejunal tissues preserved in sterile, enzyme-free freezing tubes were thawed, and total RNA was extracted from these tissues using TRIzol reagent (cat. no. 15596026CN, Thermo Fisher Scientific, USA), followed by quality control and integrity assessment of the extracted RNA. Oligo (dT) magnetic beads were used to enrich mRNA with a polyA structure in total RNA, and ion disruption was used to break the RNA into fragments of about 300 bp in length. Next, the first strand of cDNA was synthesized. Briefly, based on RNA template, 6-base random primers and reverse transcriptase were used to synthesize the first strand of cDNA, the second strand of cDNA was synthesized based on the first strand template of cDNA. Subsequently, PCR amplification was performed for library fragment enrichment. A 450 bp library was selected based on the amplified fragments. Next, the Agilent 2,100 Bioanalyzer was used to quality the library. After quality control, different Index sequences were mixed with the library in proportion. The mixed library was uniformly diluted to 2 nm and become a single chain library through alkaline denaturation. After RNA extraction, purification and library construction, the libraries were subjected to paired-end sequencing using Next-Generation Sequencing based on the Illumina sequencing platform. The reference genome used for analysis was GCF_016772045.1_ARS-UI_Ramb_v2.0_genomic.fna, which corresponds to the sheep genome. Differential expression analysis was conducted using DESeq2. We applied a |log2FC| threshold of 1.0 and an adjusted *p* value threshold of 0.05 to identify differentially expressed genes (DEGs). Each group consisted of 5 biological replicates.

### 2.7 Determination of the ROS levels

After fixation in paraformaldehyde, jejunal tissues were subjected to gradient sucrose dehydration: immersed in 15% sucrose solution for 24 h, followed by 30% sucrose solution for another 24 h. The intestinal tissues were embedded in OCT compound (cat. no. 4583, Sakura) and subsequently sectioned in a freezer sectioning machine (−20 °C) at a thickness of 10 μm. The intestinal tissues were stained with ROS staining solution (cat. no. G1045, Servicebio, China) for 30 min under light-avoidance conditions. Subsequently, the nuclei of the stained slides were restained using DAIP (cat. no. G1012, Servicebio, China). The slides were washed three times with PBS (5 min each time), dried, mounted using anti-fade medium. Fluorescence was detected using an orthogonal fluorescence microscope (Nikon ECLIPSE C1, Japan). The average optical density value of each image was quantified using ImageJ by selecting the specific fluorescent signal associated with ROS staining to ensure accurate measurement of the positive staining across all images.

### 2.8 Statistical analysis

The normality of the data distribution was assessed using the Shapiro–Wilk test, and the homogeneity of variances was evaluated using Levene’s test. Normally distributed data with homogeneous variances were analyzed using Student’s t-test, whereas non-normally distributed data were analyzed using the Wilcoxon Rank Sum test. Normally distributed data with unequal variances were analyzed using Welch’s *t*-test. All statistical analyses were performed using SPSS 24.0 software. Data are presented as mean ± SD. A *p*-value of <0.05 was considered to indicate a statistically significant difference.

## 3 Results

### 3.1 Effects of dietary sodium butyrate addition on jejunal morphology

As shown in Table 2, compared to the CON group, sodium butyrate supplementation in the diets of weaned lambs significantly increased the jejunal villus height (*p* < 0.05). However, there was no significant change in the crypt depth and villus-to-crypt ratio between the two groups. (*p* > 0.05).

**Table 2:** Effects of sodium butyrate supplementation on jejunal morphology of weaned lambs.

Items	Treatments		*p*-value
	CON	NaB	
Villus height (μm)	494.45 ± 51.97	631.63 ± 66.78	0.048
Crypt depth (μm)	283.85 ± 34.98	338.92 ± 43.31	0.162
Villus height/crypt depth (V/C)	1.75 ± 0.12	1.87 ± 0.18	0.384

### 3.2 Effects of dietary sodium butyrate addition on jejunal antioxidant indicators

As shown in Table 3, sodium butyrate supplementation in the diets at early weaning significantly increased the level of T-AOC in the jejunal tissue of lambs (*p* < 0.05). Furthermore, the content of MDA in the jejunal tissue of lambs was significantly reduced by the addition of sodium butyrate (*p* < 0.05). However, no significant differences were observed in the activities of SOD and GSH-Px between the two groups (*p* > 0.05).

**Table 3:** Effects of sodium butyrate supplementation on jejunal antioxidant indicators of weaned lambs.

Items^1^	Treatments		*p*-value
	CON	NaB	
T-AOC (U/mg)	0.81 ± 0.04	0.90 ± 0.06	0.033
SOD (U/g)	357.64 ± 23.18	350.70 ± 32.73	0.709
GSH-Px (U/g)	50.30 ± 5.15	48.99 ± 3.54	0.652
MDA (nmol/g)	24.65 ± 2.26	20.54 ± 1.87	0.014

^1^ T-AOC, total antioxidant capacity; SOD (superoxide dismutase); GSH-Px, glutathione peroxidase; MDA, malondialdehyde.

### 3.3 Effects of dietary sodium butyrate addition on the ROS levels of jejunal tissues

**Figure 1:**
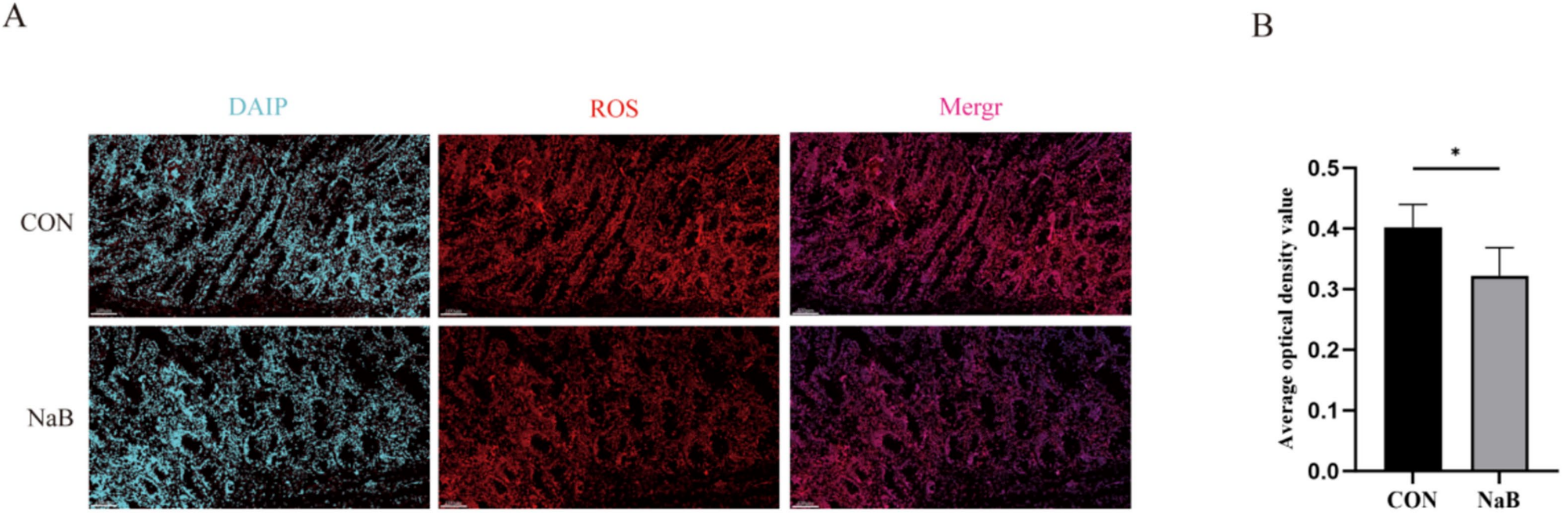
Effects of sodium butyrate supplementation on accumulation of ROS in jejunal tissues in weaned lambs. **(A)** Fluorescence intensity of ROS in jejunal tissue. **(B)** Statistical results of average optical density of ROS in the jejunal tissues of lambs.

As shown in Figure 1, compared with the CON group, dietary sodium butyrate addition significantly reduced the fluorescence intensity of ROS in the jejunal tissues of lambs (*p* < 0.05), which suggested that the addition of sodium butyrate in the diet can effectively reduce the accumulation of reactive oxygen species in the jejunal tissues of lambs.

### 3.4 Effects of dietary sodium butyrate addition on jejunal mitochondrial morphology

**Figure 2:**
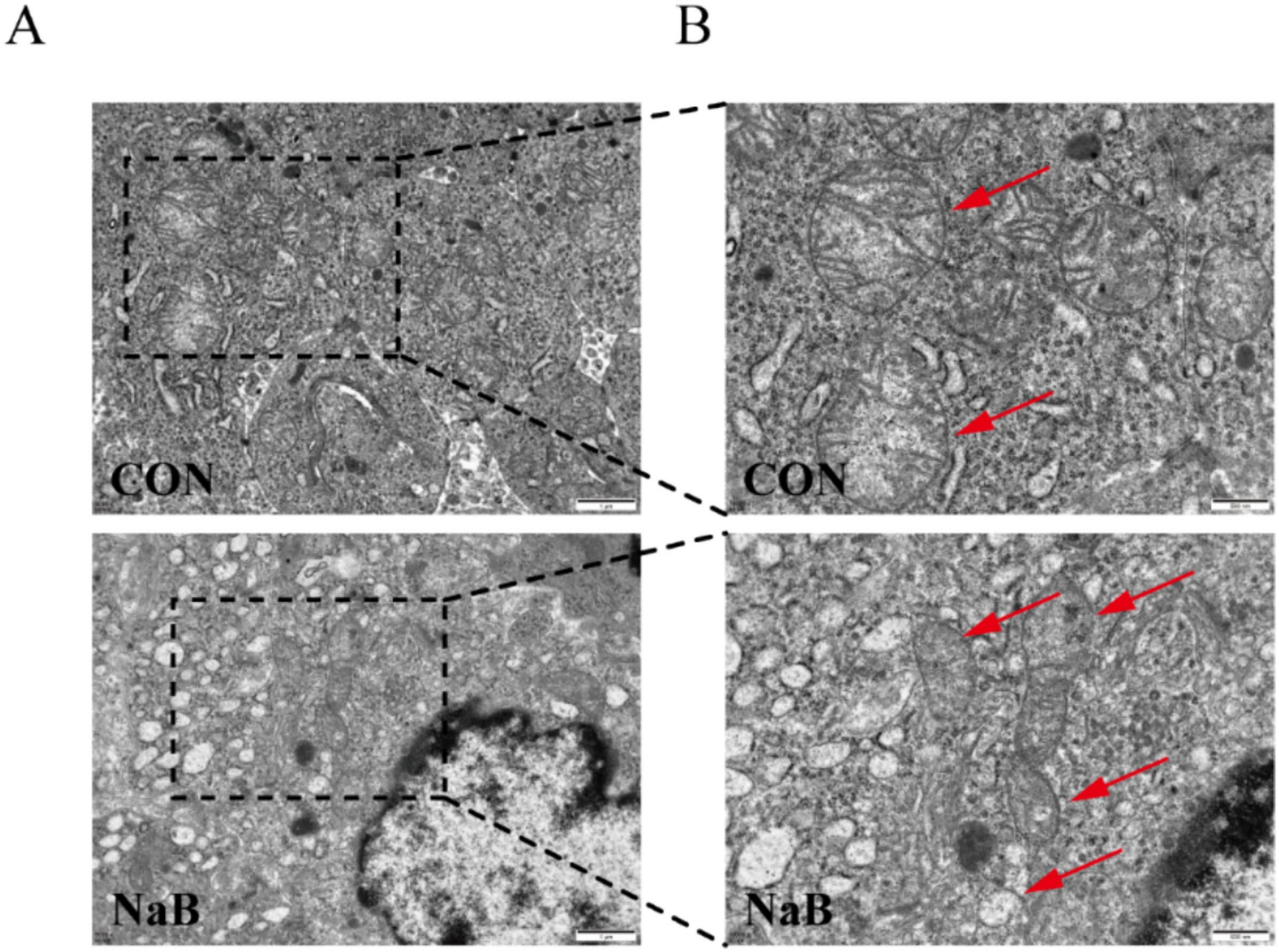
Effects of sodium butyrate supplementation on mitochondrial ultrastructure in jejunum of weaned lambs. **(A)** 5,000 × magnifications with a 1 μm ruler. **(B)** 10,000 × magnifications with a 500 nm ruler. Red arrows indicate mitochondria.

The effects of dietary sodium butyrate addition on the mitochondrial ultrastructure of jejunum in weaned lambs were shown in Figure 2. In the CON group, mitochondria exhibited fuzzy cristae and empty central matrix space. Conversely, dietary sodium butyrate supplementation showed a more complete mitochondrial morphology, and the lamb jejunal tissue mitochondria possessed well-defined cristae and intact membranes.

### 3.5 Transcriptomics profiling results

**Figure 3:**
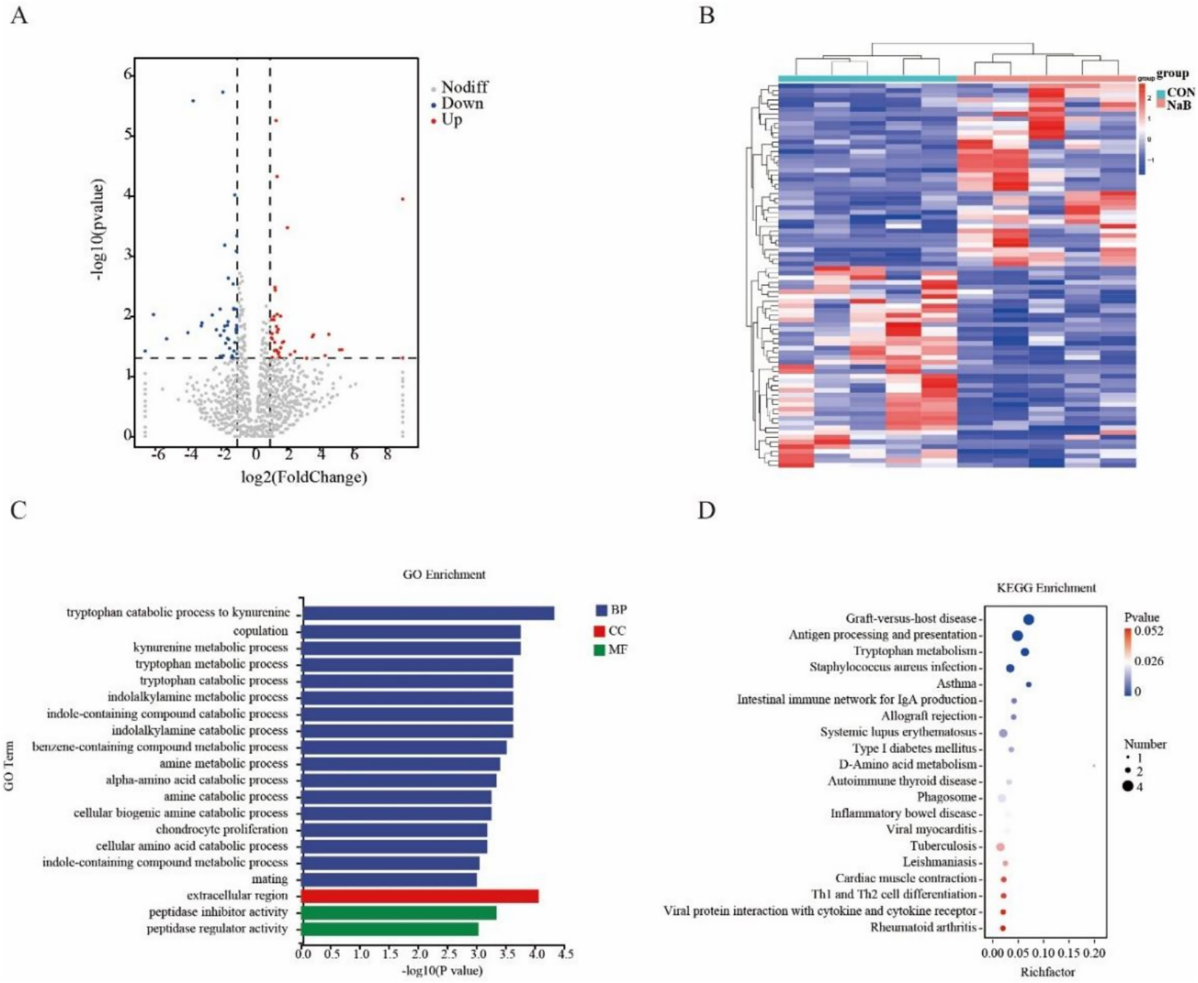
Effects of sodium butyrate supplementation on transcriptome in jejunum of weaned lambs. **(A)** The volcano map of differentially expressed genes between the CON and NaB groups (a |log_2_FC| threshold of 1.0 and an adjusted *p*-value threshold of 0.05). **(B)** The heat map of differentially expressed genes between the CON and NaB groups. **(C)** The GO enrichment of differentially expressed genes between the CON and NaB groups. **(D)** The KEGG enrichment of differentially expressed genes between the CON and NaB groups.

As shown in Figure 3A, a total of 82 differentially expressed genes (DEGs) were identified between the CON and NaB groups, including 43 up-regulated and 39 down-regulated genes in the jejunal tissues of lambs in the NaB group. The heatmap in Figure 3B further demonstrated a clear separation in jejunal gene expression patterns between the two groups. GO enrichment analysis showed that the DEGs were not randomly distributed, but were mainly enriched in several biologically meaningful categories (Figure 3C). Among the biological process terms, the most prominent enrichments were related to tryptophan catabolic process to kynurenine, kynurenine metabolic process, tryptophan metabolic process, tryptophan catabolic process, and amino acid/amine catabolic processes. In addition, the main cellular component term was extracellular region, whereas the major molecular function terms included peptidase inhibitor activity and peptidase regulator activity. These results indicate that sodium butyrate mainly affects amino acid metabolic remodeling and extracellular regulatory functions in the jejunum. Notably, the enrichment of the tryptophan–kynurenine metabolic axis is mechanistically relevant because this pathway is closely associated with intestinal immune homeostasis and may influence mitochondrial redox balance and cellular energy metabolism. KEGG enrichment analysis further identified several functionally relevant pathways, including tryptophan metabolism, antigen processing and presentation, intestinal immune network for IgA production, phagosome, and Th1 and Th2 cell differentiation (Figure 3D). These pathways suggest that dietary sodium butyrate exerts coordinated effects on metabolic adaptation and mucosal immune regulation in the jejunum. Specifically, modulation of tryptophan metabolism may contribute to the maintenance of cellular energy metabolism and antioxidant capacity, whereas regulation of antigen processing and presentation, IgA production, phagosome function, and Th1/Th2 differentiation may help alleviate inflammatory stress and thereby indirectly protect mitochondrial function and epithelial homeostasis. Overall, transcriptomic data indicate that sodium butyrate may facilitate jejunal epithelial adaptation in early-weaned lambs by coordinately regulating amino acid metabolism, immune defense, and epithelial functional stability.

## 4 Discussion

As a critical developmental stage for lambs, the weaning period affects nutrient absorption, utilization, and immune function in weaned lambs (12). Sodium butyrate-based feed supplements have attracted widespread attention due to their ability to effectively address a range of intestinal health issues in animal production (13). Additionally, sodium butyrate participates in multiple interactions involving microbiota, feed digestion, gastrointestinal integrity, and immune responsiveness (14). The impact of early weaning stress on intestinal health has been widely reported (15–17). Studies have indicated that the dietary changes can alter the gut microbiota composition and structure in lambs, consequently impacting intestinal health (18). Notably, sodium butyrate plays a critical role in maintaining intestinal epithelial integrity, effectively mitigating rumen damage caused by high-concentrate diets (19). Previous studies have suggested that sodium butyrate enhances innate immune function in the gut through G-protein-coupled receptor-mediated signaling pathways. Simultaneously, it suppresses histone deacetylase to alleviate excessive inflammatory responses, promoting intestinal health (9). Furthermore, sodium butyrate contributes to mucosal barrier repair by activating macrophages and the Wnt/Erk signaling pathway, ultimately improving gut health (20). Our research findings demonstrated that dietary supplementation with sodium butyrate significantly increased villus height in the jejunum tissues, improved jejunal morphological structure in weaned lambs. This enhancement would facilitate nutrient absorption and contributes to lamb intestinal health.

Early weaning is associated with increased oxidative stress in lambs, which may impair intestinal function. Sodium butyrate has been reported to improve antioxidant status (21). In the present study, dietary sodium butyrate supplementation significantly increased (T-AOC) and decreased (MDA) levels in the jejunal tissues of early-weaned lambs, indicating an improved overall antioxidant status and reduced lipid peroxidation. MDA is a well-recognized marker of oxidative damage to cell membranes (22); therefore, its reduction suggests that sodium butyrate may alleviate weaning stress-induced oxidative injury in the jejunum and thereby contribute to the maintenance of intestinal function. However, the activities of SOD and GSH-Px were not significantly altered, suggesting that the beneficial effect of sodium butyrate on redox status in this study was mainly reflected in enhanced total antioxidant capacity rather than changes in these individual antioxidant enzymes.

Apart from intestinal damage, the imbalance of oxidation and antioxidant capacity can trigger excessive intracellular ROS accumulation. Excess ROS directly damage mitochondrial structure and function, thus affecting the normal cellular activities (23, 24). Previous study have indicated that mitophagy eliminates damaged mitochondrial DNA, thereby maintaining mitochondrial integrity (25). Sodium butyrate not only promotes mitochondrial self-repair via the AMPK-mitophagy pathway but also upregulates key mitophagy-related proteins to alleviate mitochondrial damage induced by oxidative stress (8). Additionally, sodium butyrate enhances mitophagy by increasing the expression of COX-2, Nrf2, HO-1, and Pink1/Parkin during mitophagy initiation (26). Notably, sodium butyrate contributes to mitochondrial repair and functional recovery by reducing ROS levels, increasing mitochondrial membrane potential, and enhancing the expression of mitochondrial DNA and function-related genes (27). Based on ultrastructural observations of mitochondrial morphology and determination of ROS levels in jejunal tissues, our study demonstrated that sodium butyrate markedly reduced ROS accumulation and enhanced antioxidant capacity in early-weaned lambs. Furthermore, dietary sodium butyrate supplementation effectively maintained jejunal mitochondrial morphology. Collectively, sodium butyrate exhibits potential positive regulatory effects in mitigating and repairing oxidative stress-mediated mitochondrial damage.

As previously mentioned, dietary supplementation with sodium butyrate can alleviate mitochondrial damage induced by early-weaning stress. Mitochondria, as crucial organelles for energy metabolism, play an important role in energy utilization. Early weaning poses significant challenges to the intestinal function of young ruminants, impacting nutrient absorption in the jejunum (28). Recent research have indicated that sodium butyrate promotes intestinal development and regulates nutrient absorption (29). This beneficial effect of sodium butyrate on nutrient absorption can be attributed to its multifaceted roles in maintaining intestinal development, repairing inflammatory injury, and reshaping the gut microbiota (27). Furthermore, based on transcriptomic analysis of jejunal tissues from the sodium butyrate-treated group, our study confirmed its roles in nutrient metabolism and immune regulation within intestinal tissues. Therefore, sodium butyrate supplementation in the diets of early-weaned lambs may effectively improve jejunal epithelial structure and maintain stable metabolic function. However, the relatively small sample size is a limitation of the present study and may have reduced statistical power. Thus, these transcriptomic findings should be interpreted with caution and further validated in future studies with larger sample sizes.

## 5 Conclusion

Dietary supplementation with 3 g/kg of sodium butyrate can enhance jejunal villus height and antioxidant capacity, reduce the accumulation of ROS in the jejunum, and maintain the integrity of jejunal mitochondrial structure of early weaned lambs. Additionally, transcriptome data indicate that dietary supplementation with sodium butyrate can regulate jejunal nutrient-metabolic processes and immune functions in early weaned lambs.

## Data Availability

The datasets presented in this study can be found in online repositories. The names of the repository/repositories and accession number(s) can be found below: http://www.ncbi.nlm.nih.gov/bioproject/1131192.
